# Secreted protein acidic and rich in cysteine (SPARC) is upregulated by transforming growth factor (TGF)-β and is required for TGF-β-induced hydrogen peroxide production in fibroblasts

**DOI:** 10.1186/1755-1536-6-6

**Published:** 2013-03-21

**Authors:** Saiko Shibata, Junichi Ishiyama

**Affiliations:** 1 Discovery Research Laboratories, Kyorin Pharmaceutical Co., Ltd 2399-1 Nogi-machi, Shimotsuga-gun, Tochigi 329-0114, Japan

**Keywords:** SPARC, TGF-β, Hydrogen peroxide, Fibroblast, Pulmonary fibrosis

## Abstract

**Background:**

Idiopathic pulmonary fibrosis (IPF) is a poorly understood progressive disease characterized by the recurrent damage of alveolar epithelial cells as well as inappropriate expansion and activation of fibroblasts resulting in pronounced extracellular matrix (ECM) deposition. Although recent studies have indicated the involvement of secreted protein acidic and rich in cysteine (SPARC), a matricellular protein regulating ECM deposition, in the pathogenesis of fibrosis, factors regulating SPARC expression or roles of SPARC in fibrosis have not been fully elucidated.

**Results:**

Among the profibrotic factors examined in cultured fibroblasts, we showed that SPARC expression was upregulated mainly by transforming growth factor (TGF)-β. We also showed that expression of *SPARC* in the lung was upregulated in the murine bleomycin-induced pulmonary fibrosis model, which was inhibited by TGF-β receptor I inhibitor. Knockdown of SPARC in fibroblasts using siRNA or treatment with the antioxidant N-acetylcysteine attenuated epithelial cell injury induced by TGF-β-activated fibroblasts in a coculture system. We also demonstrated that SPARC was required for hydrogen peroxide (H_2_O_2_) production in fibroblasts treated with TGF-β. Furthermore, TGF-β activated integrin-linked kinase (ILK), which was inhibited by SPARC siRNA. Knockdown of ILK attenuated extracellular H_2_O_2_ generation in TGF-β-stimulated fibroblasts. Our results indicated that SPARC is upregulated by TGF-β and is required for TGF-β-induced H_2_O_2_ production via activation of ILK, and this H_2_O_2_ production from fibroblasts is capable of causing epithelial cell injury.

**Conclusions:**

The results presented in this study suggest that SPARC plays a role in epithelial damage in the IPF lung via enhanced H_2_O_2_ production from fibroblasts activated by TGF-β. Therefore, SPARC inhibition may prevent epithelial injury in IPF lung and represent a potential therapeutic approach for IPF.

## Background

Idiopathic pulmonary fibrosis (IPF) is a progressive and fatal lung disease of unknown etiology with a median survival of 4 to 5 years following diagnosis [1]. IPF is characterized by epithelial cell apoptosis and fibroblast proliferation resulting in pronounced extracellular matrix (ECM) deposition [2,3]. Although the pathogenesis of IPF remains incompletely understood, one of the most widely accepted views is that the recurrent damage of alveolar epithelial cells (AEC) leads to AEC apoptosis as well as inappropriate expansion and activation of fibroblasts. This aberrant fibroblast activation causes excessive ECM production and accumulation. AEC apoptosis and pronounced ECM deposition are profoundly linked to impairment of respiratory function [4,5].

Recent studies have shown that oxidative stress is one of the causes of AEC damage and apoptosis in IPF [4,6]. Reactive oxygen species (ROS) contribute to the establishment and progression of pulmonary fibrosis in animal models and possibly also in human IPF [7]. Disruption of the normal oxidant/antioxidant balance [8,9] and deficiency of antioxidants [6] have been found in the lungs and lower respiratory tract, respectively, in IPF. Furthermore, it has been shown that fibroblasts obtained from the lungs in IPF generate high ROS levels [10]. Although the mechanisms underlying the elevation of ROS in the lungs in IPF have not been elucidated in detail, recent studies have shown that TGF-β induces the production of hydrogen peroxide (H_2_O_2_) via activation of NAD(P)H oxidases in human lung fibroblasts [11,12]. TGF-β is a multifunctional cytokine that regulates not only the activity of NAD(P)H oxidases but also a variety of physiological process, including cell growth, differentiation, profibrotic gene expression, fibroblast proliferation, ECM expression, and epithelial-mesenchymal transition, and is thought to be a key regulator of progressive fibrosis [13,14].

Secreted protein acidic and rich in cysteine (SPARC) is a matricellular protein that binds directly to ECM proteins, such as collagen, and participates in ECM assembly and turnover [15]. Moreover, SPARC interacts with several integrins as well as growth factors and regulates downstream signaling pathways [16]. In recent studies, SPARC was shown to modulate downstream components of integrin signaling, such as activation of integrin-linked kinase (ILK), which plays a significant role in cell adhesion, motility and survival [17]. It has been shown that expression of SPARC is regulated by TGF-β in several types of fibroblast. It has also been reported that SPARC regulates the expression and activity of TGF-β [18]. Accumulating evidence suggests that SPARC may contribute to the progression of pulmonary fibrosis. In the bleomycin-induced pulmonary fibrosis model, SPARC-null mice show a diminished amount of pulmonary fibrosis compared to controls [19]. Fibroblasts with attenuated SPARC expression by small interfering RNA (siRNA) show reduced expression of Type I collagen. Moreover, induction of Type I collagen upon TGF-β stimulation is diminished in SPARC-knockdown fibroblasts [20]. These studies suggest that SPARC may be a key regulatory molecule in the pathogenesis of IPF. However, factors capable of regulating SPARC expression and the role of SPARC in the pathogenesis of fibrosis have not been fully elucidated. In this study, we investigated which profibrotic factors can regulate the induction of SPARC. We also examined whether SPARC contributes to H_2_O_2_ production in fibroblasts, which is linked to epithelial cell injury.

## Results

### Induction of SPARC is mainly regulated by TGF-β both *in vitro* and *in vivo*

**Figure 1:**
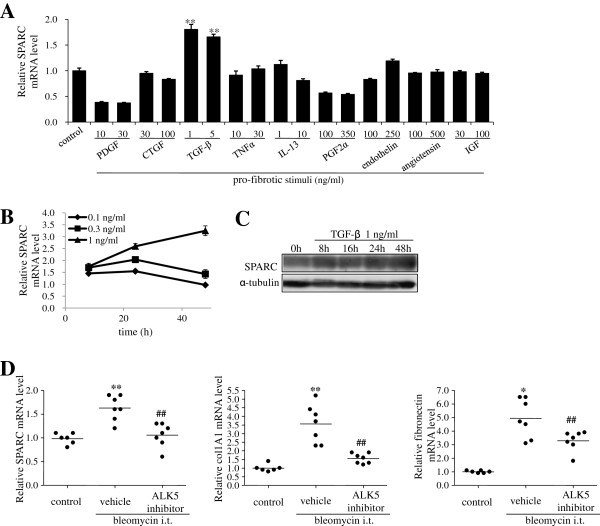
**Secreted protein acidic and rich in cysteine (SPARC) expression is induced by selective transforming growth factor (TGF)-β stimulation.** (**A**) HFL-1 cells were treated with profibrotic factors for 24 h and *SPARC* gene expression was quantified by real-time PCR and normalized relative to 18S rRNA. **P <0.01 versus control. (**B**) and(**C**) SPARC expression in HFL-1 cells stimulated with TGF-β for the indicated times at the indicated concentration. SPARC expression was analyzed by real-time PCR, and relative *SPARC* expression was calculated by comparison with the non-stimulated *SPARC* level at each time point (**B**) or by western blotting (**C**). (**D**) Total lung RNA was isolated from homogenates of lungs from mice treated with vehicle or ALK5 inhibitor, 11 days after intratracheal instillation of saline or bleomycin. *SPARC*, *Col1A1* and *Fibronectin* gene expression were quantified by real-time PCR and normalized relative to 18S rRNA. *P <0.05, **P <0.01 versus control;^#^*P* <0.01 versus vehicle. (**A**) and (**B**) represent the means of three independent experiments ± SE.

Although SPARC was reported to be upregulated by TGF-β or angiotensin II in several types of fibroblast [21,22], it has not been fully elucidated whether other factors, associated with the progression of pulmonary fibrosis, upregulate SPARC expression. Therefore, we studied SPARC gene expression in HFL-1 cells in response to the profibrotic stimuli platelet-derived growth factor (PDGF), connective tissue growth factor (CTGF), transforming growth factor (TGF)-β, tumor necrosis factor (TNF)-α, IL-13, prostaglandin F_2α_ (PGF_2α_), endothelin-1, angiotensin II, and insulin-like growth factor (IGF). Only TGF-β stimulation induced *SPARC* mRNA expression (Figure 1A). The upregulation of *SPARC* by TGF-β (1 ng/ml) was approximately 1.5-fold as early as 8 h after treatment and lasted up to 48 h (Figure 1B). SPARC protein induction was also observed 8 h after TGF-β stimulation, which continued up to 48 h (Figure 1C). To investigate whether SPARC induction is also regulated by TGF-β *in vivo*, we studied *SPARC* gene expression in a bleomycin-induced murine pulmonary fibrosis model. As reported previously by other groups, *SPARC* mRNA expression in the lung increased following intratracheal instillation of bleomycin (Figure 1D). Treatment with SB-525334, a selective inhibitor of TGF-β activin receptor-like kinases (ALK5), resulted in a significant reduction in *SPARC* mRNA expression, as well as expression of fibrotic genes, such as *Col1A1* and *Fibronectin*, in the lungs (Figure 1D). These findings suggest that SPARC induction is upregulated by TGF-β both *in vitro* and *in vivo*.

### PI3K and p38 mitogen-activated protein kinase (MAPK) signaling are involved in SPARC induction by TGF-β

**Figure 2:**
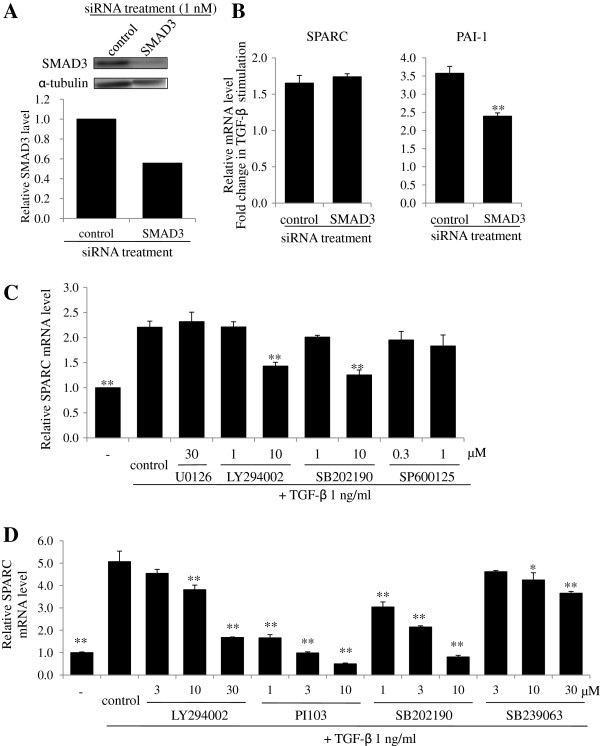
**PI3K and P38 mitogen activated protein kinase (MAPK) but not SMAD3 contribute to the induction of Secreted protein acidic and rich in cysteine (SPARC) by transforming growth factor (TGF)-β.** (**A**) HFL-1 cells were transfected with non-targeting control or SMAD3 siRNA for 24 h, starved of serum for 24 h, and then cell lysates were subjected to western blotting analysis for SMAD3 expression. Bar graph shows densitometric analysis of western blotting. (**B**) HFL-1 cells were transfected with non-targeting control or SMAD3 siRNA for 24 h, starved of serum for 24 h, and then stimulated with TGF-β (1 ng/ml) for 24 h. *SPARC* and *PAI-1* gene expression were analyzed by real-time PCR, and normalized relative to 18S rRNA. Data are expressed as means ± SE of three independent experiments. **P < 0.01 versus non-targeting control. (**C** and **D**) After 24 h of serum starvation, HFL-1 cells were stimulated with TGF-β (1 ng/ml) for 24 h in the presence/absence of the inhibitors U0126 (MEK inhibitor), LY294002, PI103 (PI3K inhibitor), SB202190, SB239063 (p38 MAPK inhibitor), or SP600125 (JNK inhibitor). *SPARC* gene expression was analyzed by real-time PCR, and normalized relative to 18S rRNA. Data are expressed as means ± SE of three independent experiments. *P <0.05, **P <0.01 versus control. MEK, mitogen-activated protein kinase kinase; JNK, c-Jun N-terminal kinase.

Although induction of SPARC by TGF-β has been demonstrated previously *in vitro*, the signaling pathway involved in this regulation has not been explored in detail. To determine which downstream signaling of TGF-β is required for SPARC expression, we used siRNA and pharmacological inhibitors. SMAD3 protein level was reduced in HFL-1 cells transfected with SMAD3 siRNA compared with control siRNA (Figure 2A). SMAD3 knockdown significantly alleviated induction of *PAI-1*, which is a gene known to be upregulated by TGF-β in a SMAD3-dependent manner. In contrast, a decrease in SMAD3 expression failed to alter *SPARC* expression (Figure 2B). TGF-β also activates non-SMAD pathways, such as mitogen-activated protein kinase kinase (MEK), p38 mitogen-activated protein kinase (MAPK), phosphoinositide 3-kinase (PI3K), and c-Jun N-terminal kinase (JNK). We used pharmacological inhibitors of these molecules (MEK inhibitor: U0126; PI3K inhibitor: LY294002; p38 MAPK inhibitor: SB202190; JNK inhibitor: SP600125) to examine the involvement in SPARC induction by TGF-β. Reasonability of the concentration of each pharmacological inhibitor was confirmed by the inhibitory effect of each inhibitor on the target kinase activity as evaluated by phosphorylation of its substrate protein (see Additional file 1). Pretreatment with LY294002 and SB202190 significantly reduced *SPARC* induction by 64% and 79%, respectively (Figure 2C). As SP600125 at concentrations exceeding 1 μM induced cell death, the involvement of JNK in *SPARC* induction by TGF-β could not be fully elucidated. To confirm the involvement of the PI3K and p38 MAPK signaling pathway in the induction of SPARC by TGF-β, we used other pharmacological inhibitors (PI3K inhibitor: PI103; p38 MAPK inhibitor: SB239063). Similar to LY294002, PI103 markedly attenuated *SPARC* expression in a concentration-dependent manner (Figure 2D). SB239063 also significantly inhibited *SPARC* expression (Figure 2D). Therefore these results indicated that PI3K and p38 MAPK are involved in TGF-β-dependent induction of SPARC in HFL-1 cells.

### SPARC siRNA prevents the epithelial cell death induced by TGF-β-stimulated fibroblasts

**Figure 3:**
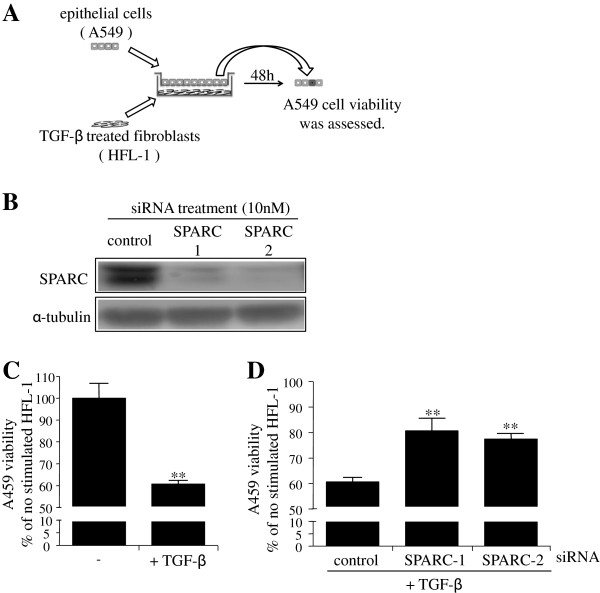
**Secreted protein acidic and rich in cysteine (SPARC) contributes to the epithelial cell death caused by transforming growth factor (TGF)-β-stimulated fibroblasts.** (**A**) Coculture system of A549 cells and TGF-β stimulated HFL-1 cells. (**B**) HFL-1 cells were transfected with non-targeting control or SPARC siRNA for 24 h, starved of serum for 24 h, and then cell lysates were subjected to western blotting analysis of SPARC expression. (**C**) HFL-1 cells pretreated with or without TGF-β (2 ng/ml) for 16 h were washed before introduction of A549 cells. A549 cell viability was assessed following 48 h of coculture by Cell Counting kit-8. Data are expressed as means ± SE of three independent experiments. **P <0.01 versus TGF-β (−). (**D**) HFL-1 cells transfected with non-targeting control or SPARC siRNA were pretreated with or without TGF-β (2 ng/ml) for 16 h, and then washed before introduction of A549 cells. A549 cell viability was assessed following 48 h of coculture by Cell Counting kit-8. Data are expressed as means ± SE of three independent experiments. **P <0.01 versus non-targeting control siRNA.

Apoptosis of type II AEC is a well known characteristic of the lung in IPF [2,4]. It has been reported that lung epithelial cells overlying TGF-β-stimulated fibroblasts obtained from the lungs in IPF show increased rates of cell death [23], suggesting that activated fibroblasts are capable of damaging epithelial cells. Therefore, we investigated whether SPARC contributes to epithelial injury caused by TGF-β-activated fibroblasts. For this purpose, we used the compartmentalized coculture system [23]. HFL-1 cells were grown in the lower wells of the Transwell coculture system and A549 cells were grown on permeable membranes in the upper chambers with removable inserts (Figure 3A). Both cell types were seeded and cultured independently before coculture. HFL-1 cells were stimulated with TGF-β for 16 h and then washed to remove TGF-β before introduction of inserts containing A549 cells. HFL-1 cells and A549 cells were cocultured for 48 h, and then A549 cell viability was determined using a Cell Counting Kit-8. As reported previously, TGF-β-stimulated HFL-1 cells reduced A549 cell viability (Figure 3C). Following successful downregulation of SPARC at the protein level with two different types of SPARC siRNA transfection (Figure 3B), we found that knockdown of SPARC in HFL-1 cells restored the loss of A549 cell viability induced by TGF-β-stimulated HFL-1 cells (Figure 3D).

### SPARC siRNA inhibits H_2_O_2_ release from HFL-1 cells following TGF-β stimulation

**Figure 4:**
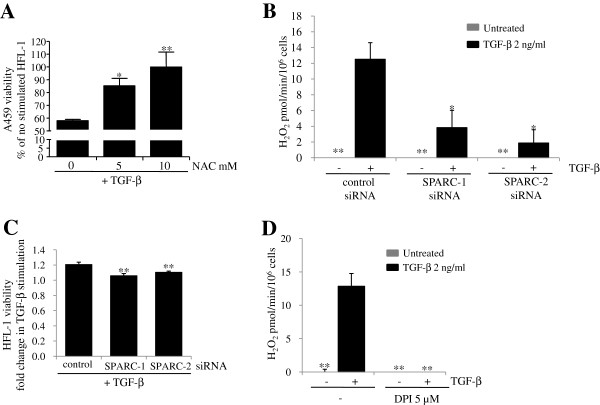
**Secreted protein acidic and rich in cysteine (SPARC) knockdown attenuates H**_**2**_**O**_**2 **_**release from HFL-1 following transforming growth factor (TGF)-β stimulation.** (**A**) HFL-1 cells pretreated with or without TGF-β (2 ng/ml) for 16 h were cocultured with A549 cells for 48 h in the absence or presence of NAC. A549 cell viability was assessed following 48 h of coculture by Cell Counting Kit-8. Data are expressed as means ± SE of three independent experiments. *P <0.05, **P <0.01 versus NAC 0 mM. (**B**) HFL-1 cells transfected with non-targeting control or SPARC siRNA were treated with or without TGF-β (2 ng/ml) for 16 h before H_2_O_2_ measurements. Data are expressed as means ± SE. Three replicate experiments showed similar results. **P <0.01 versus TGF-β-stimulated HFL-1 transfected with non-targeting control siRNA. (**C**) HFL-1 cells transfected with non-targeting control or SPARC siRNA were pretreated with or without TGF-β (2 ng/ml) for 16 h, and then washed before introduction of A549 cells. HFL-1 cell viability was assessed following 48 h of coculture by Cell Counting kit-8. Data are expressed as means ± SE of three independent experiments. **P <0.01 versus TGF-β-stimulated HFL-1 transfected with non-targeting control siRNA. (**D**) HFL-1 cells pretreated with or without TGF-β (2 ng/ml) for 16 h in the absence or presence of diphenyliodonium (DPI) before H_2_O_2_ measurements. Data are expressed as means ± SE. Three replicate experiments showed similar results. **P < 0.01 versus TGF-β-stimulated HFL-1 in the absence of DPI.

Next, we attempted to elucidate how SPARC contributes to epithelial cell death induced by TGF-β-stimulated fibroblasts. As SPARC is a secreted protein, SPARC induced by TGF-β from HFL-1 cells may affect the A549 cell viability. Therefore, we treated A549 cells with SPARC for 48 h. However, we found that SPARC by itself did not affect A549 cell viability (data not shown). We then examined whether SPARC has an influence on factors reducing A549 cell viability secreted from HFL-1 cells upon stimulation with TGF-β. As H_2_O_2_ secreted by IPF fibroblasts has been shown to induce death of small AEC [23], we added N-acetylcysteine (NAC), which is a ROS scavenger, to the compartmentalized coculture system. After 48 h of coculture, NAC treatment completely prevented the loss of A549 cell viability induced by TGF-β-stimulated HFL-1 cells (Figure 4A). This result suggested that ROS, such as H_2_O_2_, secreted from HFL-1 cells may evoke the loss of A549 cell viability. To examine whether H_2_O_2_ can contribute to the loss of A549 cell viability, we added H_2_O_2_ into the Transwell coculture system of A549 cells and the SPARC-knockdown HFL-1 cells. We found that exogenously applied H_2_O_2_ negated prevention of the loss of A549 cell viability by SPARC knockdown (see Additional file 2). Therefore, HFL-1 cells were stimulated with TGF-β for 16 h and extracellular H_2_O_2_ production was measured. There was no measurable release of H_2_O_2_ from unstimulated HFL-1 cells (Figure 4B). Elevated H_2_O_2_ was detected after 16 h of TGF-β stimulation (Figure 4B). We then examined the possible role of SPARC in this H_2_O_2_ production. After successful downregulation of SPARC by RNA interference (Figure 3B), we found that SPARC deficiency significantly abolished TGF-β-induced H_2_O_2_ production by HFL-1 cells (Figure 4B). To avoid the possibility that SPARC deficiency depletes HFL-1 cells itself rather than inhibiting H_2_O_2_ production, we assayed HFL-1 cell viability with Cell Counting Kit-8 under coculture conditions. SPARC deficiency only marginally affected viability (Figure 4C). H_2_O_2_ secretion by TGF-β-stimulated HFL-1 cells was completely abolished by treatment with diphenyliodonium (DPI), which is an inhibitor of flavoenzymes such as NAD(P)H oxidases (Figure 4D). Our findings indicated that SPARC plays a major role in H_2_O_2_ secretion induced by TGF-β via NAD(P)H oxidases. Because it is known that TGF-β upregulates NADPH oxidase 4 (NOX4) in a variety of cell types [24], we examined the contribution of NOX4 to the H_2_O_2_ secretion by TGF-β. Knockdown of NOX4 using siRNA almost completely abolished H_2_O_2_ secretion by TGF-β (see Figure S3 A and B in Additional file 3), suggesting that NOX4 is a major NADPH oxidase contributing to TGF-β-stimulated H_2_O_2_ production in HFL-1 cells. Therefore, we studied whether SPARC contributes to NOX4 upregulation by TGF-β. As a result, SPARC knockdown partially reduced NOX4 expression (see Figure S3 C in Additional file 3).

### SPARC promoted H_2_O_2_ release following TGF-β stimulation through ILK activation

**Figure 5:**
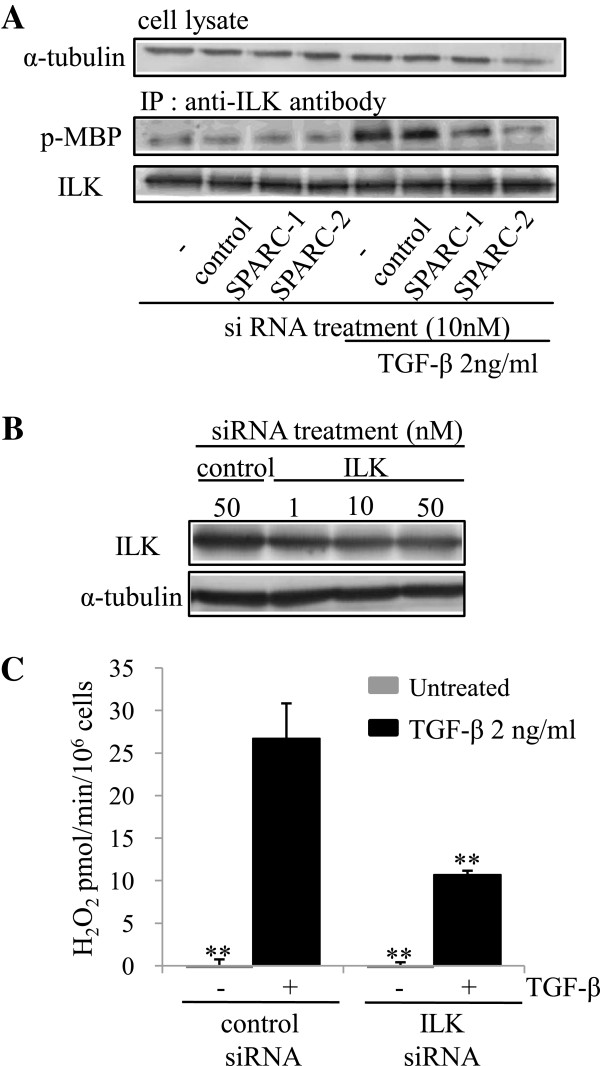
**Integrin-linked kinase (ILK) activation by Secreted protein acidic and rich in cysteine (SPARC) requires H**_**2**_**O**_**2**_** release following transforming growth factor (TGF)-β stimulation in HFL-1 cells.** (**A**) HFL-1 cells transfected with non-targeting control or SPARC siRNA were treated with or without TGF-β (2 ng/ml) for 16 h and then cell lysates were subjected to ILK assay. ILK activity was assayed by western blotting analysis of phospho-myelin base protein (MBP) level. (**B**) HFL-1 cells were transfected with non-targeting control or ILK siRNA for 24 h, starved of serum for 24 h, and then cell lysates were subjected to western blotting analysis for ILK expression. (**C**) HFL-1 cells transfected with non-targeting control or ILK siRNA were treated with or without TGF-β (2 ng/ml) for 16 h before H_2_O_2_ measurements. Data are expressed as means ± SE. Three replicate experiments showed similar results. **P <0.01 versus TGF-β-stimulated HFL-1 cells transfected with non-targeting control siRNA.

To determine the molecular mechanism by which SPARC promotes H_2_O_2_ secretion by TGF-β, we examined the involvement of ILK in this process because ILK activation was shown to be associated with pro-survival activity of SPARC in lens epithelial cells [17]. To measure ILK activity, ILK protein was immunoprecipitated and the degree of phosphorylation of Myelin basic protein (MBP) was assessed as ILK activity. After 16 h of TGF-β treatment, ILK activation was observed as determined by phosphorylated MBP, which was diminished by SPARC knockdown (Figure 5A). Our results indicated that SPARC is required for ILK activation induced by TGF-β. We used ILK siRNA to examine whether SPARC-related ILK activation contributes to H_2_O_2_ production. ILK protein level was reduced by about 50% in HFL-1 cells transfected with ILK siRNA (Figure 5B). ILK knockdown alleviated induction of H_2_O_2_ by TGF-β in HFL-1 cells by approximately 40% (Figure 5C). As we obtained only partial knockdown of ILK protein, we were unable to determine whether complete inhibition of ILK could diminish H_2_O_2_ production completely. However, our results suggested that ILK activation is at least partially involved in SPARC-mediated H_2_O_2_ secretion by TGF-β.

## Discussion

IPF is a chronic, progressive parenchymal lung disease for which no effective therapy has yet been developed. A better understanding of the molecular mechanisms underlying the pathogenesis and progression of the disease is required for the development of novel therapeutic regimens for IPF. Recent studies suggested a significant contribution of SPARC to the pathogenesis of pulmonary fibrosis. However, the roles of SPARC have not been fully elucidated. In the present study, we demonstrated that SPARC enhances H_2_O_2_ production in fibroblasts treated with TGF-β.

Consistent with our observations, deletion of the SPARC gene significantly reduces the levels of urinary and renal reactive oxygen species, inflammation, and tubulointerstitial fibrosis in angiotensin II-infused mice [22]. It is well known that increased ROS levels can cause epithelial cell apoptosis in culture [25,26]. Moreover, activated myofibroblasts, which produce significant amounts of extracellular ROS, are sufficient to induce apoptosis of adjacent epithelial cells [23]. Alveolar epithelial injury is considered to be one of the main characteristics of the lung in IPF, and recurrent epithelial damage is thought to cause fibrotic changes, and eventually result in fatal respiratory dysfunction [2,4]. Inhibition of ROS production by NOX4 gene deletion [27,28] and administration of the radical scavenger NAC [29] were shown to have protective effects against alveolar epithelial injury in the bleomycin-induced lung fibrosis model. A recent clinical trial indicated that NAC monotherapy may have some beneficial effects in the early stages of IPF although it failed to significantly change forced vital capacity [30]. These reports indicated that elevated ROS production is one of the causative factors of recurrent epithelial damage in fibrotic lungs. Therefore, SPARC may be involved in epithelial cell injury through enhanced H_2_O_2_ production from activated fibroblasts. This hypothesis is supported by our results indicating that knockdown of SPARC expression level by siRNA mitigated the decrease in viability of A549 epithelial cells in coculture with TGF-β-stimulated fibroblasts. This reduction in A549 cell viability was alleviated in the presence of NAC. In addition, interference with SPARC expression by siRNA reduced H_2_O_2_ release from fibroblasts treated with TGF-β. SPARC has been shown to play an important role in ECM accumulation [15,31]. In addition to this role of SPARC in the pathogenesis of fibrosis, our findings indicated a possible contribution of SPARC to epithelial cell damage through regulation of ROS production.

We demonstrated the involvement of ILK in the mechanism underlying enhanced ROS production by SPARC, which was supported by a number of observations. First, knockdown of SPARC with siRNA diminished ILK activation in TGF-β-stimulated fibroblasts. Second, siRNA against ILK significantly reduced extracellular H_2_O_2_ generation in TGF-β-stimulated fibroblasts. Our findings were consistent with those of previous studies indicating that SPARC activates ILK in fibroblasts [32] and that activation of ILK by high pressure leads to ROS production in vessels through Rac-1-mediated NAD(P)H oxidase activation [33]. In isolated cardiomyocytes, ILK is activated by stromal cell-derived factor 1 (SDF-1) and is necessary for SDF-1-triggered activation of Rac-1, NAD(P)H oxidase, and release of ROS [34]. ILK interacts with the cytoplasmic domain of the integrin β1/β3 subunits, which is important for cell adhesion, differentiation, and survival [35]. Blocking of SPARC-integrin β1 interaction by function-blocking anti-integrin β1 antibody impairs ILK activation [17], suggesting that SPARC-ILK signaling is mediated at least in part by integrin β1.

NADPH oxidase family of proteins is comprised of five members, including NADPH oxidase 1 to 5 [36]. In the present study, knockdown of NOX4 using siRNA almost completely blocked TGF-β-induced H_2_O_2_ production in HFL-1 cells (see Figure S3 B in Additional file 3), suggesting NOX4 is a major NADPH oxidase involved in TGF-β-induced H_2_O_2_ production. It has been known that NOX4 is a constitutively active NADPH oxidase isoform and NOX4 activity is regulated, at least in part, at the transcriptional level [24]. NOX4 expression is increased by TGF-β stimulation in fibroblasts [28,37]. Consistent with these reports, our study showed that NOX4 was upregulated by TGF-β in HFL-1 cells. While knockdown of SPARC prominently blocked H_2_O_2_ production induced by TGF-β stimulation, upregulation of NOX4 expression was reduced only moderately by SPARC knockdown (see Figure S3 C in Additional file 3), implying that SPARC may promote H_2_O_2_ production through regulation of NOX4 activity rather than regulation of transcriptional level of NOX4. Although activity of NOX4 is known to be regulated at the transcriptional level, more recently several reports have shown that NOX4 activity can be regulated by the mechanisms other than transcriptional regulation. P22phox and polymerase DNA-directed delta-interacting protein 2 (poldip2) modulate NOX4 activity [24,38]. Post-translational modifications of NOX4, such as glycosylation, sumoylation or phosphorylation, are reported to be required for NOX4 activation [24,39,40]. In order to understand the precise mechanisms underlying enhancement of H_2_O_2_ production by SPARC, further studies are needed.

Another important finding in the present study was that *SPARC* expression is upregulated by TGF-β but not other profibrotic factors, such as PDGF, CTGF, TNF-α, IL-13, PGF_2α_, endothelin-1, angiotensin II, and IGF, in HFL-1 cells. In the bleomycin-induced lung fibrosis model, blocking of TGF-β signaling by the ALK-5 inhibitor SB-525334 significantly decreased *SPARC* expression as well as the degree of fibrosis. These results suggest that SPARC may be selectively upregulated by TGF-β and promote fibrotic changes via ROS production and ECM deposition. In accordance with our results, several previous studies indicate that TGF-β increases SPARC expression at both mRNA and protein levels in gingival fibroblasts, dermal fibroblasts, and pulp cells [21,41]. In contrast to our results, angiotensin II was reported to increase SPARC level in renal mesangial cells [22]. Thus, SPARC expression may be regulated by different factors in a cell type-specific manner. Although previous studies demonstrated regulation of SPARC by TGF-β, the signaling pathway involved in this regulation has not been explored in detail. In the present study, we showed that p38 MAPK and PI3K signaling are important for SPARC induction by TGF-β rather than the SMAD3 pathway using pharmacological inhibitors and siRNA experiments.

TGF-β signals are transduced by transmembrane Type I and Type II serine/threonine kinase receptors, which phosphorylate transcriptional factors SMAD2 and SMAD3. TGF-β also uses non-SMAD signaling pathways, such as MEK, PI3K-AKT, p38 MAPK, and JNK [42]. We examined whether TGF-β activates PI3K-AKT, and p38 MAPK in HFL-1 cells. We found that TGF-β treatment induced AKT phosphorylation within 20 minutes (data not shown). On the other hand, p38 MAPK was phosphorylated in the basal state. Both AKT and p38 MAPK phosphorylation were reduced in the presence of specific inhibitors of these pathways. Our observations indicated that the basal activity of p38 MAPK and TGF-β-induced PI3K-AKT activation are involved in SPARC induction. With regard to the importance of PI3K and p38 MAPK in the pathogenesis of fibrosis, it was shown that phosphorylated AKT is strongly expressed in areas of pulmonary fibrosis after intratracheal administration of bleomycin in mice, and that blockade of PI3K-AKT signaling attenuates pulmonary fibrosis induced by bleomycin treatment or TGF-β overexpression [43,44]. It has also been reported that inhibition of p38 MAPK attenuates the progression of fibrosis in the bleomycin model [45]. SPARC may serve as one of the downstream factors of PI3K and p38 MAPK signaling in the pathogenesis of fibrosis. Although PDGF is also known to be able to activate both PI3K and p38 MAPK signalling pathways [46,47], SPARC upregulation was not induced by PDGF stimulation in our study. Therefore, activation of PI3K and p38 MAPK is required but is not enough for SPARC induction. Other signaling pathways could also be involved in upregulation of SPARC by TGF-β.

## Conclusions

Our results indicated that SPARC contributes to the extracellular H_2_O_2_ generation induced by TGF-β via ILK activation in fibroblasts and can regulate the viability of adjacent epithelial cells through H_2_O_2_ generation. In addition, SPARC expression is upregulated by TGF-β, which is thought to be a key regulator for the establishment and progression of IPF, not only in culture but also in the animal model of pulmonary fibrosis. One of the most widely accepted views regarding the pathogenesis of IPF is the recurrent damage of alveolar epithelial cells and ECM deposition from aberrant activated fibroblasts [2,3]. We demonstrated that SPARC likely contributes to epithelial damage through regulation of ROS production. As SPARC is capable of exerting pleiotropic functions on the pathogenesis of IPF, SPARC inhibition may represent a potential therapeutic approach for IPF.

## Methods

### Materials

TGF-β, PDGF, IL-13 and IGF were purchased from R&D systems (Minneapolis, MN, USA). CTGF and TNFα were purchased from Pepro Tech (Rocky Hill, NJ, USA). Endothelin-1 and angiotensin II were purchased from Sigma-Aldrich (St. Louis, MO, USA). PGF_2α_ was purchased from Enzo life science (Farmingdale,NY, USA). Antibody against SPARC was purchased from Santa Cruz Biotechnology (Santa Cruz, CA, USA). Antibodies against SMAD3, α-Tubulin, p-p44/42(Thr202/Tyr204), p44/42, p-AKT (Ser473), AKT, p-c-Jun (Ser63), c-Jun, p-p38 MAPK (Thr180/Tyr182), p38 MAPK and ILK were purchased from Cell Signaling Technology (Billerica, MA, USA). Antibody against ILK was purchased from Abnova (Taipei city, Taiwan). Phospho MBP was purchased from Milipore (Billerica, MA, USA). U0126, LY294002, PI103, SB202190, SB239063 and SP600125 were purchased from Calbiochem (San Diego, CA, USA). Diphenyliodonium (DPI) and N-acetylcysteine (NAC) were purchased from Sigma-Aldrich.

### Cell culture

The human fetal lung fibroblast HFL-1 and the human lung adenocarcinoma epithelial cell line A549 were obtained from the American Type Culture Collection (Manassas, VA, USA) and maintained in DMEM (Life Technologies, Carlsbad, CA, USA) supplemented with 10% FBS and 100 U/ml penicillin/streptomycin (P/S) at 37°C under 5% CO_2_. Studies were performed on passage 5 to 10 of HFL-1 cells.

### Coculture system of epithelial cells and fibroblasts

HFL-1 cells were plated on the lower wells of 24-well transwell co-culture system at a density of 1 ×10^5^ cells/well, and cultured at 37°C under 5% CO_2_ for overnight. Then cells were grown for 24 h in DMEM with 0.5% FBS before treatment with/without TGF-β. After 16 h, HFL-1 cells were washed twice with PBS before insertion of the upper chambers, which contained A549 cells plated the day before at a density of 1 ×10^4^ cells/upper chamber, in the transwell coculture system. After 48 h coculture, the cell viability was assessed by measuring mitochondrial succinate dehydrogenase activity using Cell counting Kit-8 (Kumamoto, Dojindo, Japan) according to the manufacturer’s instructions.

### Measurement of H_2_O_2_ release

H_2_O_2_ release from cultured HFL-1 cells into the overlying medium was measured by coupling horseradish peroxidase (HRP) activity using the conversion of Amplex red to resorufin in the presence of H_2_O_2_ as described previously [23]. At 16 h of exposure of TGF-β, all cells were washed with PBS, and then incubated with the reaction mixture containing 100 μM Amplex red, 5 U/ml HRP, and 1mM 4-(2-hydroxyethyl)-1-piperazineethanesulfonic acid (HEPES) in Hank's Balanced Salt Solution (HBSS) without phenol red, pH 7.4. This solution was collected following 90-minute incubation, and fluorescence was measured at excitation and emission wavelengths of 544 nm and 590 nm, respectively. The exact H_2_O_2_ concentrations of solutions were calculated by standard curves plots.

### Real-time PCR

Total RNA from HFL-1 cells was isolated using a Qiagen RNeasy mini kit (Qiagen, Valencia, CA, USA) according to the manufacturer’s instructions. For mice lung tissue, total RNA was extracted using TRIzol (Life Technologies) and purified with Qiagen RNeasy mini kit. RNA was reverse-transcribed using a high-capacity cDNA reverse transcription kit (Applied Biosystems, Foster City, CA, USA). Quantitative gene expression analysis was performed by real-time PCR on an AB7500 fast real-time PCR system (Applied Biosystems) using TaqMan gene expression assay of SPARC (Hs00234160_m1, Mm00486332_m1), Col1A1 (Mm00801666), Fibronectin (Mm01256744_m1), PAI-1 (Hs00167155_m1) and NOX4 (Hs01558199_m1). The 18 rRNA (Hs99999901_s1) was amplified in the same reaction to act as reference.

### Transfection of SPARC, SMAD3 and ILK siRNA

HFL-1 cells were transfected with Stealth Select RNAi (Life Technologies) directed against SPARC (HSS110131, HSS110133), SMAD3 (HSS106252), ILK (HSS140843) and NOX4 (HSS121312, HSS121313) using Lipofectamine RNAiMAX transfection reagent (Life Technologies). Stealth RNAi Negative Control Duplex was used as a non-targeting control. Following 48-h incubation, the efficiency of siRNA knockdown of endogenous SPARC, SMAD3, ILK or NOX4 was assayed by western blotting analysis or real-time PCR.

### ILK assay

HFL-1 cells transfected with non-targeting control or SPARC siRNA were treated with or without TGF-β for 16h and then cell lysate (200 μl) was mixed with rabbit monoclonal anti-ILK antibody (Cell Signaling Technology, Beverly, MA, USA) and Protein A/G-Sepharose (GE Healthcare, Piscataway, NJ, USA). Complexes were washed with ILK kinase buffer (50 mM HEPES, 5 mM Na_3_VO_4_, 5 mM NaF, 10 mM MgCl_2_ and 2 mM MnCl_2_). For ILK activity assay, samples were incubated at 30°C for 25 minutes in ILK kinase buffer containing 400 μM ATP and 10 μg/ml MBP (HyTest, Turku, Finland). Complexes were analyzed by western blotting for phosphorylated MBP.

### Western blotting analysis

Cells were washed with ice-cold PBS, then lysed in cold radioimmunoprecipitation assay (RIPA) buffer containing Complete Protease Inhibitor Cocktail (Roche, Basel, Switzerland). Protein concentration was measured using the BCA protein assay reagent kit (Thermo Scientific Pierce, Rockford, IL, USA). The cell lysates were then subjected to SDS-PAGE followed by western Blotting. Antigen-antibody complexes were detected using an appropriate alkaline phosphatase-labeled secondary antibody with the Dychrome™ detection system (Life Technologies) according to the manufacturer’s protocol. The resulting bands were analyzed densitometrically using ImageQuant software (GE Healthcare, Piscataway, NJ, USA).

### Bleomycin-induced lung fibrosis

Specific pathogen-free male, 8-week-old imprinting control region (ICR) mice were randomly distributed into three experimental groups: 1) vehicle + saline; 2) vehicle + bleomycin; 3) ALK5 inhibitor (SB-525334) 30 mg/kg + bleomycin. SB-525334 was administered orally twice a day from the day of the intratracheal instillation of bleomycin up to the last day of the experiments. Mice were given bleomycin sulfate (Nippon Kayaku, Tokyo, Japan) in 0.8 mg/kg by intratracheal delivery under inhalation anesthesia. Mice in group 1 received saline alone. Mice were sacrificed at 11 days after bleomycin instillation. Lung tissues were collected and then immediately frozen in liquid nitrogen. All animal procedures used in this study were conducted according to the guidelines of the Institutional Animal Care and Use Committee of Discovery Research Laboratories of Kyorin Pharmaceutical Co., Ltd.

### Statistical analysis

Statistical comparisons were made using one-way analysis of variance (ANOVA) followed by Dunett’s test. For multiple comparisons, data were analyzed by one-way ANOVA followed by Tukey’s multiple comparison test. *P*<0.05 was considered statistically significant. All analyses were performed with GraphPad Prism 4 software package (GraphPad Software, San Diego, CA, USA).

## Abbreviations

AEC: alveolar epithelial cells; ALK: activin receptor-like kinase; ANOVA: analysis of variance; CTGF: connective tissue growth factor; DMEM: Dubecco’s modified Eagle’s serum; DPI: diphenyliodonium; ECM: extracellular matrix; FBS: fetal bovine serum; HBSS: Hank's Balanced Salt Solution; HEPES: 4-(2-hydroxyethyl)-1-piperazineethanesulfonic acid; HRP: horseradish peroxidase; ICR: imprinting control region; IGF: insulin-like growth factor; IL-13: interleukin-13; ILK: integrin-linked kinase; IPF: Idiopathic pulmonary fibrosis; JNK: c-Jun N-terminal kinase; MBP: myelin basic protein; MEK: mitogen-activated protein kinase kinase; NAC: N-acetylcysteine; NOX4: NADPH oxidase 4; p38 MAPK: p38 mitogen-activated protein kinase; PBS: phosphate-buffered saline; PCR: polymerase chain reaction; PDGF: platelet-derived growth factor; PGF2α: prostaglandin F_2α_; PI3K: phosphoinositide 3-kinase; poldip2: polymerase DNA-directed delta-interacting protein 2; ROS: reactive oxygen species; SDF-1: stromal cell-derived factor 1; siRNA: small interfering RNA; SMAD3: SMAD family member 3; SPARC: secreted protein acidic and rich in cysteine; TGF-β: transforming growth factor beta; TNFα: tumor necrosis factor alpha.

## Competing interests

The authors declare that they have no competing interests.

## Authors’ contributions

SS carried out the experiments and data analysis. JI and SS designed the experiments, interpreted the data and wrote the final manuscript. Both authors read and approved the final manuscript.

## Supplementary Material

Additional file 1: Figure 1 The inhibitory effect of each inhibitor on the target kinase. HFL-1 cells were stimulated with TGF-β (1 ng/ml) for 24 h in the presence/absence of the inhibitors U0126 (MEK inhibitor), LY294002, PI103 (PI3K inhibitor), SB202190, SB239063 (p38MAPK inhibitor), or SP600125 (JNK inhibitor). The inhibitory effect of each inhibitor on the target kinase activity was evaluated by phosphorylation of its substrate protein, (A)p44/42, (B)AKT, (C)c-Jun, (D)p38, by western blotting. TGF-β, transforming growth factor beta; MEK, Mitogen-activated protein kinase kinase; p38 MAPK, p38 mitogen activated protein kinase; JNK, c-Jun N-terminal kinase. Click here for file

Additional file 2: Figure 2 Effect of exogenously applied H_2_O_2_ on prevention of the loss of A549 cell viability by Secreted protein acidic and rich in cysteine (SPARC) knockdown. HFL-1 cells transfected with nontargeting control or SPARC siRNA were pretreated with or without TGF-β (2 ng/ml) for 16h, and then washed before introduction of A549cells. A549 cell viability was assessed following 48h of coculture with/without H_2_O_2_ by Cell Counting kit-8. Data are expressed as means ± SE of three independent experiments. Click here for file

Additional file 3: Figure 3 NOX4 knockdown attenuates H_2_O_2_ release from HFL-1 cells following TGF-β stimulation. (A) HFL-1 cells were transfected with non-targeting control or NOX4 siRNA for 24 h, starved of serum for 24 h, and then *NOX4* gene expression was analyzed by real-time PCR analysis, and normalized relative to 18 rRNA. Data are expressed as means ± SE of three independent experiments. **P < 0.01 versus non-targeting control. (B) HFL-1 cells transfected with non-targeting control or NOX4 siRNA were treated with or without TGF-β (2 ng/ml) for 16 h before H_2_O_2_ measurements. Data are expressed as means ± SE of three independent experiments. **P < 0.01 versus TGF-β-stimulated HFL-1 transfected with non-targeting control siRNA. (C) HFL-1 cells were transfected with non-targeting control or SPARC siRNA for 24 h, starved of serum for 24 h, and then stimulated with TGF-β (2 ng/ml) for 24 h. *NOX4* gene expression was analyzed by real-time PCR, and normalized relative to 18S rRNA. Data are expressed as means ± SE of three independent experiments. **P < 0.01 versus TGF-β-stimulated HFL-1 transfected with non-targeting control siRNA. NOX4, NADPH oxidase 4; TGF-β, transforming growth factor beta; PCR, polymerase chain reaction. Click here for file
