# Supplementary material for: Secreted protein acidic and rich in cysteine (SPARC) is upregulated by transforming growth factor (TGF)-β and is required for TGF-β-induced hydrogen peroxide production in fibroblasts
Source: Fibrogenesis Tissue Repair. 2013 Mar 21;6:6. doi: 10.1186/1755-1536-6-6 (PMC3610252; doi:10.1186/1755-1536-6-6)
Supplement: Additional file 1: Figure 1 — The inhibitory effect of each inhibitor on the target kinase. HFL-1 cells were stimulated with TGF-β (1 ng/ml) for 24 h in the presence/absence of the inhibitors U0126 (MEK inhibitor), LY294002, PI103 (PI3K inhibitor), SB202190, SB239063 (p38MAPK inhibitor), or SP600125 (JNK inhibitor). The inhibitory effect of each inhibitor on the target kinase activity was evaluated by phosphorylation of its substrate protein, (A)p44/42, (B)AKT, (C)c-Jun, (D)p38, by western blotting. TGF-β, transforming growth factor beta; MEK, Mitogen-activated protein kinase kinase; p38 MAPK, p38 mitogen activated protein kinase; JNK, c-Jun N-terminal kinase. [file 1755-1536-6-6-S1.pdf]

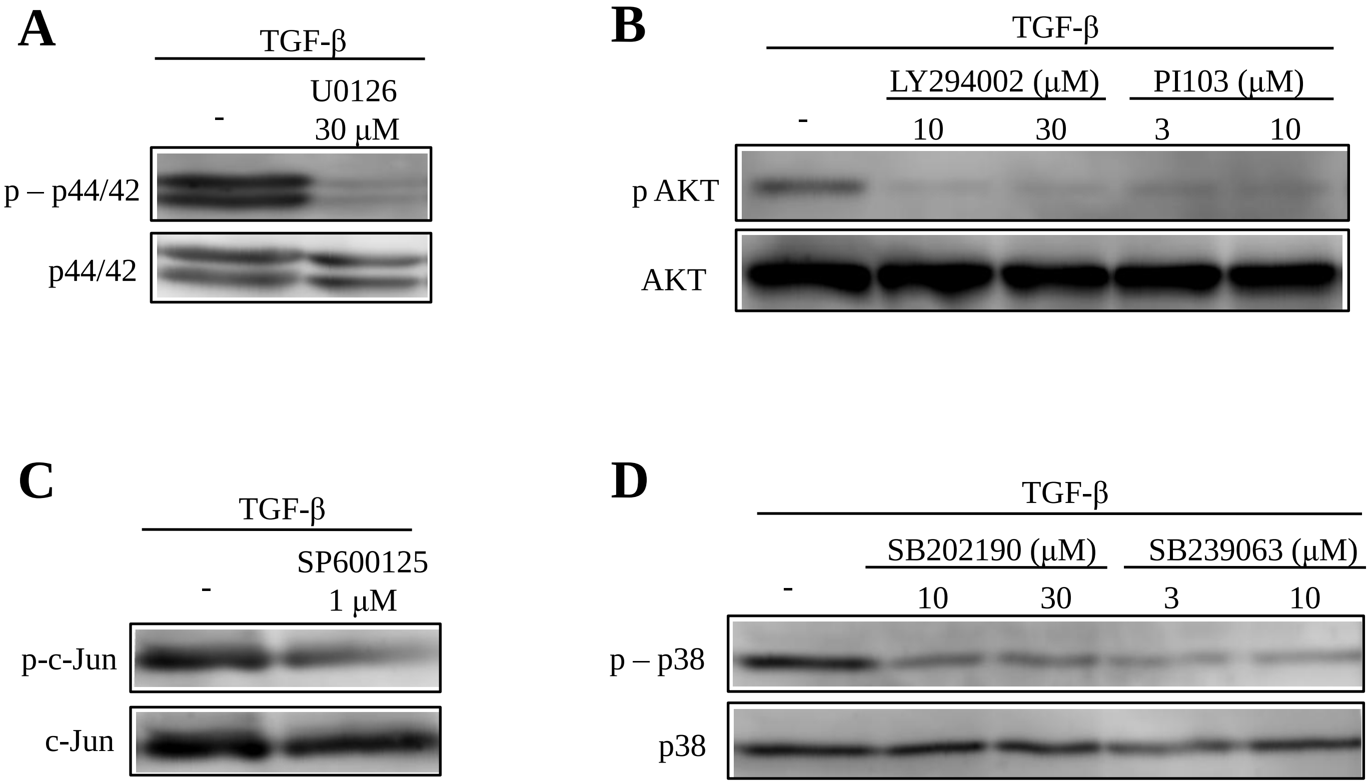


**Supplementary Figure 1 - The inhibitory effect of each inhibitor on the target kinase:**
HFL-1 cells were stimulated with TGF-β (1 ng/ml) for 24 h in the presence/absence of the inhibitors U0126 (MEK inhibitor), LY294002, PI103 (PI3K inhibitor), SB202190, SB239063 (p38 MAPK inhibitor), or SP600125 (JNK inhibitor). The inhibitory effect of each inhibitor on the target kinase activity was evaluated by phosphorylation of its substrate protein (A) p44/42 (B) AKT (C) c-Jun (D) p38 by Western blotting.
