# Supplementary material for: Secreted protein acidic and rich in cysteine (SPARC) is upregulated by transforming growth factor (TGF)-β and is required for TGF-β-induced hydrogen peroxide production in fibroblasts
Source: Fibrogenesis Tissue Repair. 2013 Mar 21;6:6. doi: 10.1186/1755-1536-6-6 (PMC3610252; doi:10.1186/1755-1536-6-6)
Supplement: Additional file 2: Figure 2 — Effect of exogenously applied H2O2 on prevention of the loss of A549 cell viability by Secreted protein acidic and rich in cysteine (SPARC) knockdown. HFL-1 cells transfected with nontargeting control or SPARC siRNA were pretreated with or without TGF-β (2 ng/ml) for 16h, and then washed before introduction of A549cells. A549 cell viability was assessed following 48h of coculture with/without H2O2 by Cell Counting kit-8. Data are expressed as means ± SE of three independent experiments. [file 1755-1536-6-6-S2.pdf]

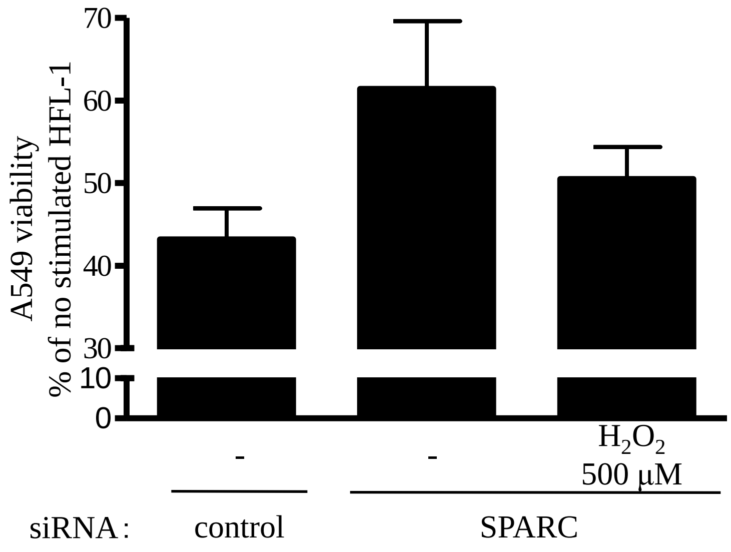

Supplementary Figure 2 - Effect of exogenously applied H_2O_2 on prevention of the loss of A549 cell viability by SPARC knockdown:

HFL-1 cells transfected with non-targeting control or SPARC siRNA were pretreated with or without TGF-β (2 ng/ml) for 16 h, and then washed before introduction of A549 cells. A549 cell viability was assessed following 48 h of coculture with/without H_2O_2 by Cell Counting kit-8. Data are expressed as means ± SE of three independent experiments.
