# Supplementary material for: Secreted protein acidic and rich in cysteine (SPARC) is upregulated by transforming growth factor (TGF)-β and is required for TGF-β-induced hydrogen peroxide production in fibroblasts
Source: Fibrogenesis Tissue Repair. 2013 Mar 21;6:6. doi: 10.1186/1755-1536-6-6 (PMC3610252; doi:10.1186/1755-1536-6-6)
Supplement: Additional file 3: Figure 3 — NOX4 knockdown attenuates H2O2 release from HFL-1 cells following TGF-β stimulation. (A) HFL-1 cells were transfected with non-targeting control or NOX4 siRNA for 24 h, starved of serum for 24 h, and then NOX4 gene expression was analyzed by real-time PCR analysis, and normalized relative to 18 rRNA. Data are expressed as means ± SE of three independent experiments. **P < 0.01 versus non-targeting control. (B) HFL-1 cells transfected with non-targeting control or NOX4 siRNA were treated with or without TGF-β (2 ng/ml) for 16 h before H2O2 measurements. Data are expressed as means ± SE of three independent experiments. **P < 0.01 versus TGF-β-stimulated HFL-1 transfected with non-targeting control siRNA. (C) HFL-1 cells were transfected with non-targeting control or SPARC siRNA for 24 h, starved of serum for 24 h, and then stimulated with TGF-β (2 ng/ml) for 24 h. NOX4 gene expression was analyzed by real-time PCR, and normalized relative to 18S rRNA. Data are expressed as means ± SE of three independent experiments. **P < 0.01 versus TGF-β-stimulated HFL-1 transfected with non-targeting control siRNA. NOX4, NADPH oxidase 4; TGF-β, transforming growth factor beta; PCR, polymerase chain reaction. [file 1755-1536-6-6-S3.pdf]

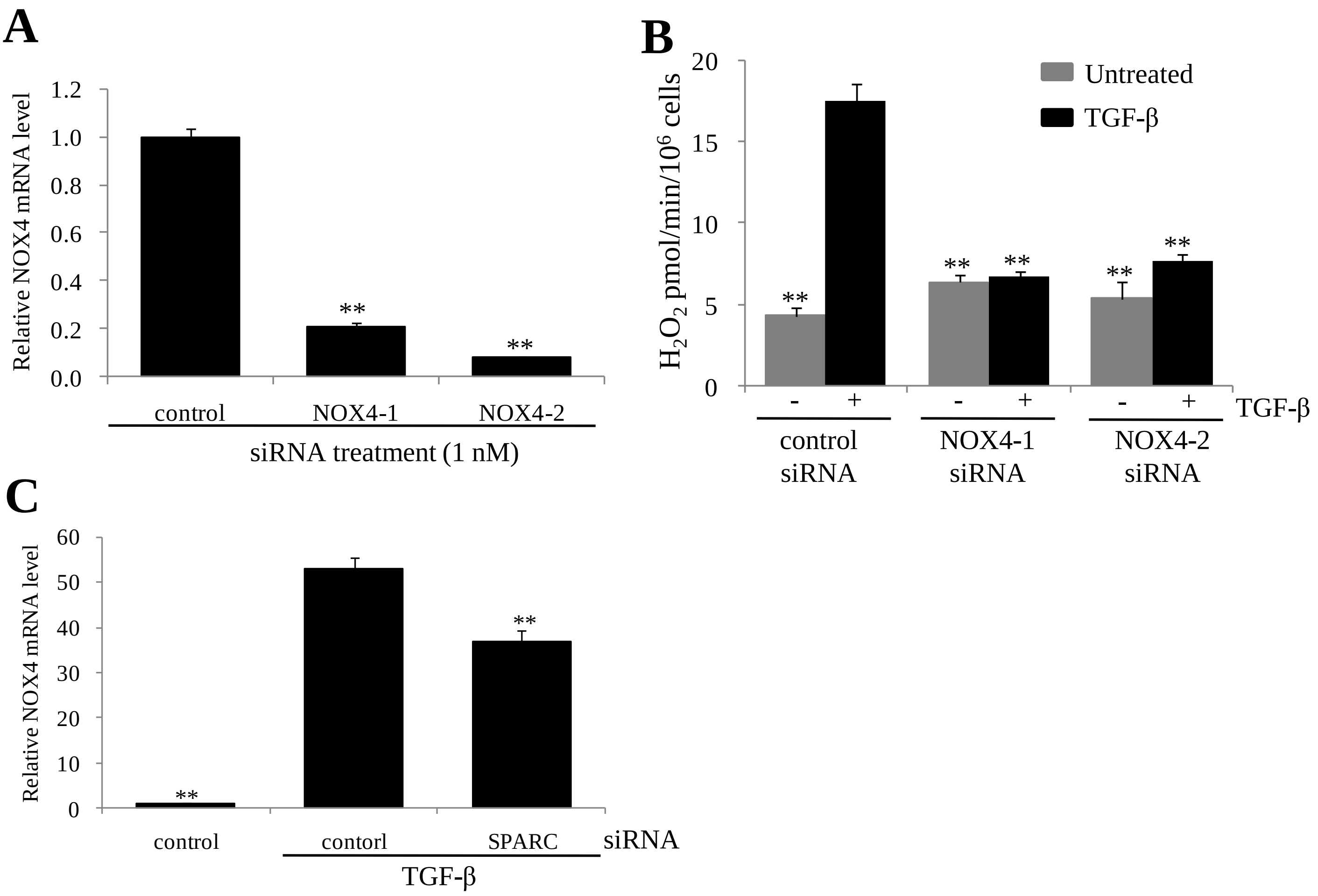

Supplementary Figure 3 - NOX4 knockdown attenuates H_2O_2 release from HFL-1 cellls following TGF-β stimulation:

(A) HFL-1 cells were transfected with non-targeting control or NOX4 siRNA for 24 h, starved of serum for 24 h, and then NOX4 gene expression was analyzed by real-time PCR analysis, and normalized relative to 18 rRNA. Data are expressed as means ± SE of three independent experiments. $^{**}P < 0.01$ versus non-targeting control. (B) HFL-1 cells transfected with non-targeting control or NOX4 siRNA were treated with or without TGF-β (2 ng/ml) for 16 h before H_2O_2 measurements. Data are expressed as means ± SE of three independent experiments. $^{**}P < 0.01$ versus TGF-β stimulated HFL-1 transfected with non-targeting control siRNA. (C) HFL-1 cells were transfected with non-targeting control or SPARC siRNA for 24 h, starved of serum for 24 h, and then stimulated with TGF-β (2 ng/ml) for 24 h. NOX4 gene expression was analyzed by real-time PCR, and normalized relative to 18S rRNA. Data are expressed as means ± SE of three independent experiments. $^{**}P < 0.01$ versus TGF-β stimulated HFL-1 transfected with non-targeting control siRNA.
